# *Crepidium* sect. *Crepidium* (Orchidaceae, Malaxidinae)—Chemical and Morphological Study of Flower Structures in the Context of Pollination Processes

**DOI:** 10.3390/plants10112373

**Published:** 2021-11-04

**Authors:** Hanna B. Margońska, Małgorzata Kozieradzka-Kiszkurno, Emilia Brzezicka, Łukasz P. Haliński, Kevin L. Davies, Monika M. Lipińska

**Affiliations:** 1 Department of Plant Taxonomy and Nature Conservation, Faculty of Biology, University of Gdańsk, Wita Stwosza 59, 80-308 Gdańsk, Poland; hanna.b.margonska@biol.ug.edu.pl; 2 Department of Plant Cytology and Embryology, Faculty of Biology, University of Gdańsk, Wita Stwosza 59, 80-308 Gdańsk, Poland; malgorzata.kozieradzka-kiszkurno@ug.edu.pl (M.K.-K.); emilia.brzezicka@ug.edu.pl (E.B.); 3 Department of Environmental Analysis, Faculty of Chemistry, University of Gdańsk, Wita Stwosza 63, 80-308 Gdańsk, Poland; lukasz.halinski@ug.edu.pl; 4 School of Earth and Environmental Sciences, Cardiff University, Cardif CF10 3AT, UK; kevinldavies@btinternet.com

**Keywords:** biofluorescence, gas chromatography, histochemistry, orchids, scanning electron microscopy, transmission electron microscopy, UV2a

## Abstract

*Crepidium* is a large genus of mainly pantropical orchids. The lips of its flowers are upwardly directed and do not serve as landing platforms for pollinators. This role is assumed by the dorsal sepal and/or gynostemium. Information about the pollination and floral morphology of this genus is scarce. To date, no papers have been published on these topics. Field observations have revealed that the flowers are visited by small flies, midges, fruit flies, other small dipterans, ants, spiders, and mites. Preliminary observations revealed at least two forms of small liquid droplets secreted on the lip surface of *Crepidium* species: simple secretions from epidermal cells, and cell sap released upon the rupturing of raphide-producing cells. Further research revealed that this was the first time liquid secretion was recorded in this genus. Floral secretions were subjected to sequential organic solvent extraction and gas chromatography–mass spectrometry (GC–MS). Floral parts were investigated by means of scanning (SEM) and transmission electron microscopy (TEM), and histochemical tests. The presence of liquid droplets on the lip of *Crepidium*, the presence of a food reward, and the sequence of raphide development are reported here for the first time.

## 1. Introduction

In terms of adaptations to different pollination syndromes, orchids are recognized as one of the most advanced plant families [1]. These adaptations may include simple changes in flower shape or color, but also more sophisticated modifications, such as imitating the shape and fragrance of a female bee (pseudocopulation; e. g. *Ophrys* L. [2]) or mimicking the fruiting body and scent of a fungus (e.g., *Dracula* Luer [3]).

**Figure 1:**
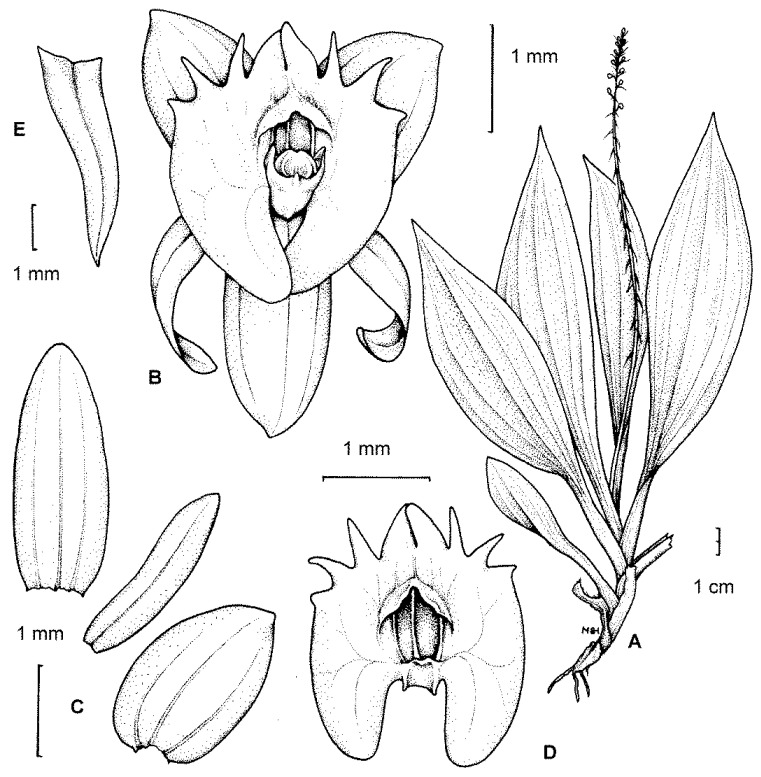
The type-species for the section *Crepidium*. *C. rheedii* Bl. (**A**)—habit; (**B**)—flower; (**C**)—tepals; (**D**)—lip; (**E**)—floral bract (drawn by H. B. Margońska, in Margońska et al., 2012 (2013)).

*Crepidium* (Bl., Bijdr. Fl. Ned. Ind. 387. 1825. *emend*. Szlach., Fragm. Flor. Geobot., Suppl. 3: 123. 1995.) is a genus of ca. 200 species which are distributed in southeast Asia and Australasia. The type-species for the type-section is *C. rheedii* Bl. (Bijdr. 387. 1825.). This section contains nearly 90 species [1] (Figure 1), and its representatives are usually terrestrial, sometimes epiphytic or lithophytic plants that generally form fairly numerous and scattered colonies. Their rhizomes are elongated (thus, the plants are well-spaced; e.g., *C. distans* (Schltr.) Szlach.) or abbreviated (plants clustered; e.g., *C. resupinatum* (G. Forst.) Szlach.), and are sometimes branched. Pseudobulbs are erect, elongate, fusiform, few-noded, usually completely covered by leaf bases and basal scales, and arise from the basal nodes of the previous pseudobulb, or from the tip of the rhizome. The leaves are usually 3–6 in number (rarely 2; rarely up to 10), spirally arranged, and clustered at the apical part of the shoot. The leaf-sheath and petiole are well-developed. The leaf-blade is always plicate and membranous; and often at least slightly oblique, ovate to lanceolate, or elliptic to oblong. The leaves are more or less attenuate, acute at the apex, basally cordate, rounded, or more rarely cuneate; and 1, 3, 5, or 7-veined. The flowers are small to medium-sized (mostly ca. 0.5 cm, occasionally up to 2.3 cm in diameter—e.g., *C*. *megalanthum* (Schltr.) Szlach.). The dorsal sepal is usually more or less oblong, elliptic, and ovate to obovate, whereas the lateral sepals are oblique, ovate, broadly ovate to nearly orbicular, and rarely oblong. Petals are linear, lanceolate, oblanceolate, with one (rarely more) nerve.

**Figure 2:**
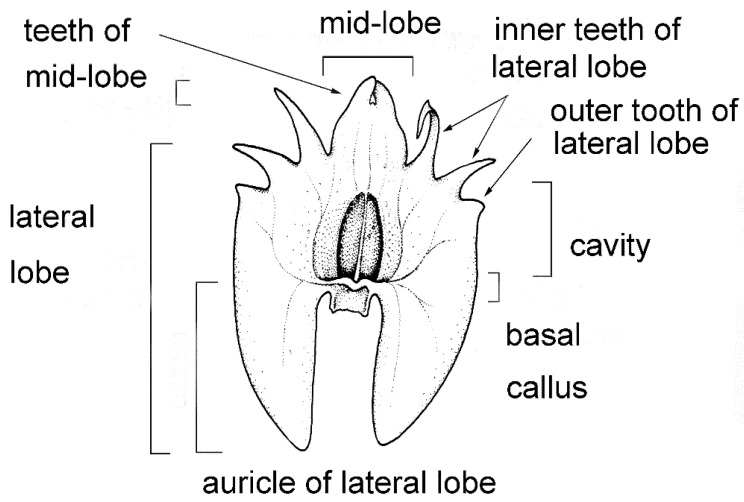
Lip morphology in the section *Crepidium*. *C. rheedii* Bl. (drawn by Margońska, in Margońska et al., 2012 (2013)).

**Figure 3:**
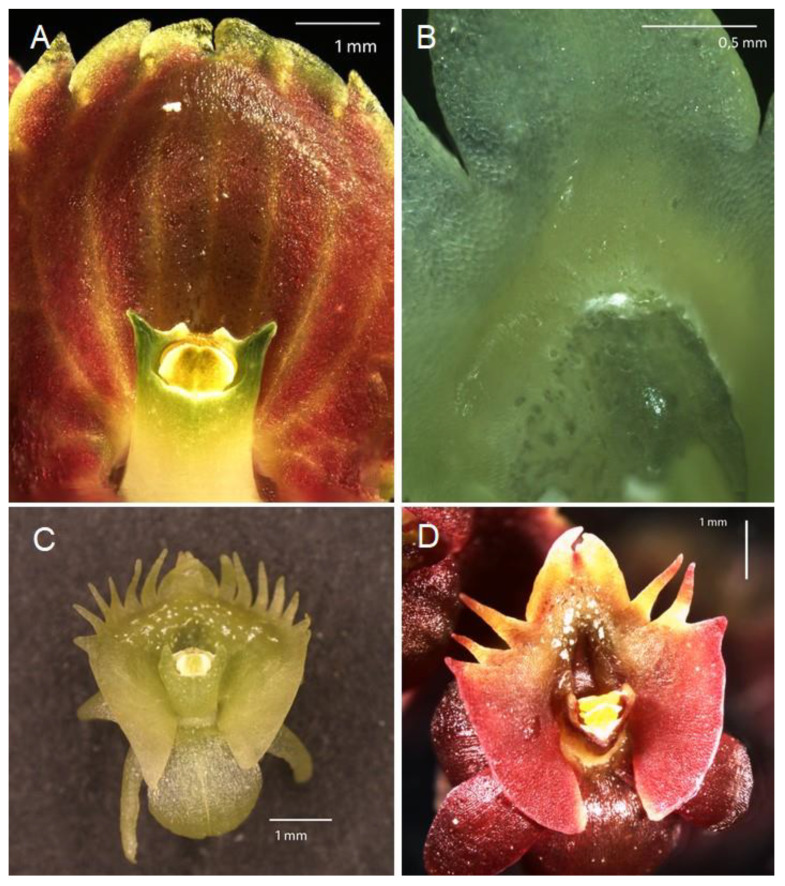
Lips of different *Crepidium* species. (**A**)—upper part of the lip of *C. metallicum*; (**B**)—fold over the lip cavity in *C.* *acutangulum*; (**C**)—autogamy by self-positioning of pollinia in *C. resupinatum*; (**D**)—fresh flower of *C. hoi* (images, H. B. Margońska).

The labellum or lip is always very distinctly 3-lobed and hippocrepiform in general outline (Figure 2). The mid-lobe is well-developed, occupying about up to one-third of the lamina length, retuse or divided at the apex, seldom entire or rarely 3-toothed (e.g., *C. atrosanguineum* (Ames) Marg. & Szlach.), and always clearly separated from the lateral lobes by a constriction or indentation. The distal margins of the lateral lobes are straight or obliquely rounded and always toothed, the teeth shorter or longer than those at the tip of the mid-lobe (e.g., *C. arachnoideum* (Schltr.) Szlach). Each of the external teeth can be developed to a greater or lesser degree. The auricles are usually distinctly longer than the gynostemium base. The cavity (sometimes referred to as the pseudonectary) is located on the central part of the lip and is surrounded by at least a slightly convex rim (e.g., *C. metallicum* (Rchb. f.) Szlach.; Figure 3A). The lip lamina usually has additional ornamentation above the cavity: a broad and convex strip, a broad glabrous or papillose fold, or a thickening often forming a transverse semi-lunate roof (which covers the cavity) (e.g., *C. acutangulum* (Hook. f.) Szlach.; Figure 3B). The gynostemium is of moderate size, and very uniform for the entire genus. The column is erect, elongate, gently arched, and at least twice as long as the anther. Staminodes are erect, exceeding the anther in size, oblong, and obtuse to weakly truncate at their apices. The rostellum is erect, thin, and truncate to rounded at the apex. The stigma is transversely elliptic, concave, and located in a deep pocket (which opens transversely). The anther base is located above the stigma and is erect, dorsiventrally flattened, and broadly ovate to nearly cordate. Its connective is narrow and thin, whereas both loculi open ventrally. The pollinia are bright yellow, are four per flower arranged in two pairs that vary slightly in size and shape, are generally clavate and flattened, and are completely concealed within loculi. Viscidia are thin and delicate, lamellate, viscid, and joined apically to the pollinia.

Age-related changes to the floral morphology are a common feature of representatives of Malaxidinae and Liparidinae Lindl. *ex* Miq. [1]. With time, their sepals and petals become reflexed, making the lip more prominent. Many mechanisms aim to prevent self-pollination in the orchid flower, as it is not beneficial and is considered to be evolutionarily disadvantageous. According to various estimations, self-pollination occurs in 3% [4] to 5–20% [5] of orchid species. However, in some groups of orchids, modifications of the pollination system enable autogamy when suitable pollinators are scarce or absent, and/or when the plant is subject to adverse growing conditions. Autogamy has been confirmed for approximately 30% of *Crepidium* species [1]. The positioning of the pollinia is due to their rotation through about 180° whilst still being anchored by their viscidia. This process can be induced, e.g., by gently tapping the flower, but also by the drying out of the viscidium while it is still attached to the apex of the rostellum [1] (Figure 3C).

In natural habitats, small flies and other small dipterans (e.g., Culicidae: *Culex* spp. or *Sciaridae:* *Bradysia* spp., Lycoriella spp., Sciara spp.) have been observed visiting *Crepidium* flowers on several occasions [1]. In cultivation, insects rarely show an interest in flowers of *Crepidium* (Margońska’s personal observation). However, spiders and their cobwebs that often occur on the inflorescences clearly indicate that sufficient insects visit to support these arachnids (Comber and Margońska, personal observation).

A characteristic feature of many species of *Crepidium* is the age-related change in flower color from bright to dark—they become more intensely colored (e.g., *C. resupinatum, C. metallicum*) and/or redder or more purplish (e.g., *C. taurinum* (Rchb. f.) Szlach.). This also seems to play an important role in signaling the state of flower readiness to potential pollinators. The “fully colored” flower has both a fully developed anther (including pollinia) and a stigma. Most visits by pollinators were observed in these fully colored, mature flowers, whereas the youngest and oldest flowers were ignored (Margońska in situ and ex situ observations).

The fragrances of some *Crepidium* flowers are sometimes described as resembling those of cucumbers or sweet cucumbers [1,6,7]. In our opinion, the reason for this is the presence of coumarin compounds in the tissues of these plants, and these are released as a result of, e.g., mechanical damage, however minor [1]. No obvious scent detectable by humans has been recorded for fresh flowers of most species of *Crepidium.* However, scents easily detected by humans occur in several species (e.g., the honey-like scent of *C. luniferum* (J.J. Sm.) Szlach. (sect. *Crepidium*); Margońska in situ and ex situ observations, 2019–2021).

Of the several dozen *Crepidium* species observed in situ and/or cultivated in the greenhouses of the University of Gdańsk (Poland), all produced various liquid secretions on the lip surface. For this study we chose a representative group of species from sect. *Crepidium* (which includes the type species for the genus), including *C. acutangulum*, *C. hoi* (P. O’Byrne) Marg. (Figure 3D), *C. luniferum*, *C. rheedii*, *C. resupinatum*, *C. metallicum*, *C. tixieri* (Seidenf.) Szlach., and *C. taurinum*. Further research revealed that this was the first time that floral liquid secretion was recorded in this genus. To support our observations, we also investigated the floral parts and their role in pollination by means of scanning (SEM) and transmission (TEM) electron microscopy, histochemistry, and floral autofluorescence.

## 2. Results

As in most species of Malaxidinae and Liparidineae, the flower colors of the investigated species change with age. Flowers of *C. hoi*, *C. metallicum*, *C. resupinatum*, *C. rheedii*, *C. tixieri*, and *C. taurinum* become more intensely colored with time. During anthesis, flowers of *C. acutangulum* and *C. luniferum* change from the initial greenish color, (buds and very young flowers) to white or whitish (full bloom), and finally, at senescence, whitish yellow to bright yellow. Species with white and very pale flowers (e.g., *C. acutangulum*, *C. luniferum*, and *C. tixierii*) are an exception with regard to their presence in certain habitats and/or readiness for effective pollination. We were not able to correlate the occurrence of such pale-flowered populations with more shady and humid habitats. Instead, they tended to occur in more open habitats, whereas flowers of populations occurring in shady and humid habitats were often brightly colored and therefore more visible to pollinators (observations by Comber, Margońska and Lipińska) [1].

**Figure 4:**
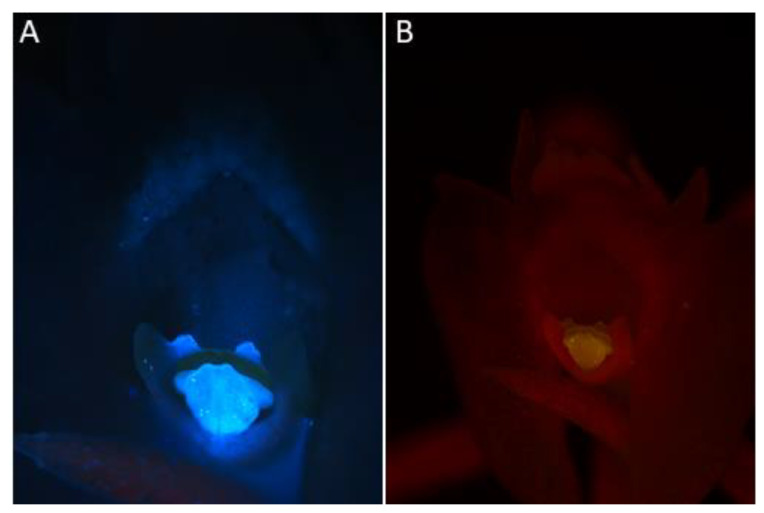
Observations of *C. acutangulum* flowers subjected to different wavelengths of light. (**A**)—Cavity of the lip and gynostemium—reflection of light with UV-2A filter (blue fluorescence); (**B**)—Cavity of the lip and gynostemium—reflection of blue light (green fluorescence) (images, M. Kapusta).

Observations of *Crepidium* flowers confirmed:

- Under UV light (Nikon UV-2A filter, excitation 330–380 nm, blue fluorescence)—soft reflection of light was observed over the entire surface of the flower, but was reflected particularly strongly by the anther, viscidia, living idioblasts with raphides, and raphides dispersed when idioblasts were damaged (Figure 4A)
- Under blue light (Nikon B-2A filter, long pass emission, excitation 450–490 nm, green fluorescence)—light was reflected particularly strongly by the anther, living idioblasts with raphides, and raphides dispersed when idioblasts were damaged (Figure 4B)
- Under green light (Nikon G-2A filter, excitation 510–590 nm, red fluorescence)—light was strongly reflected only by the anther.

We recorded, for the first time, the presence of small droplets on the lip surfaces of fresh flowers of *Crepidium*. These were located on the apical part of the central cavity (pseudonectary), and in adjacent parts, the apical half of the lip—in particular, just above the central cavity (pseudonectary) (Figure 3).

### 2.1. Scanning Electron Microscopy (SEM)

#### 2.1.1. Sepals

**Figure 5:**
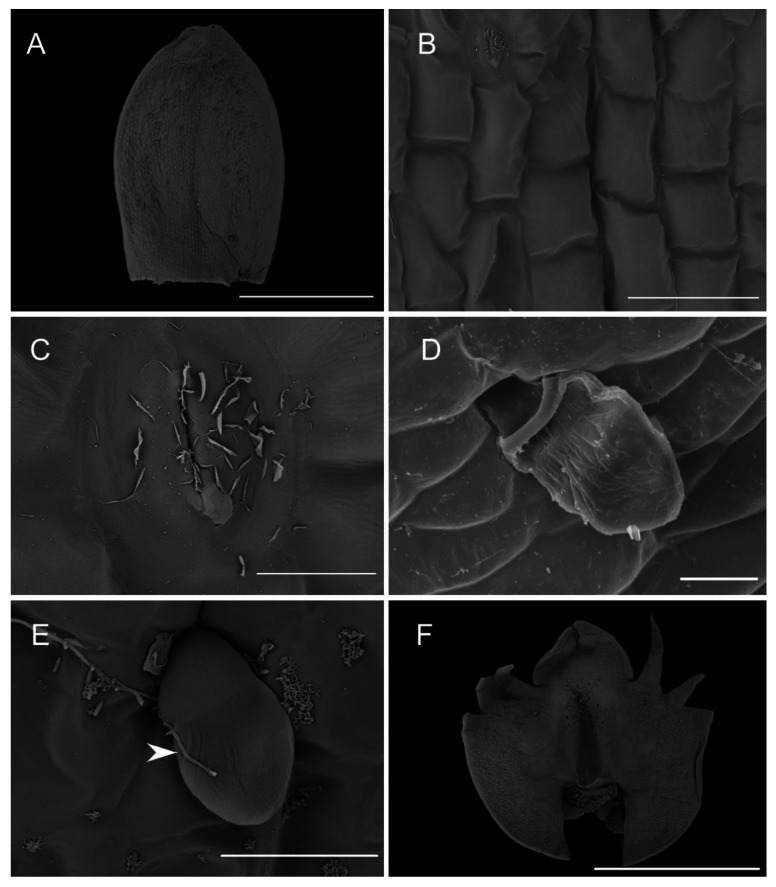
Microstructure of the dorsal sepal and lip. (**A**)—dorsal sepal of *C. hoi* (scale bar: 1 mm); (**B**)—close up of the adaxial surface of dorsal sepal of *C. hoi* (scale bar: 50 µm); (**C**)—stomata on the adaxial surface of dorsal sepal of *C. hoi* (scale bar: 10 µm); (**D**)—trichome on the adaxial surface of the dorsal sepal of *C. taurinum* (scale bar: 20 µm); (**E**)—trichome on the adaxial surface of the dorsal sepal of *C. hoi* (arrow indicates fungal hyphae; scale bar: 30 µm); (**F**)—adaxial surface of the labellum of *C. hoi* (scale bar: 2 mm). Images A-C, E-F, H. B. Margońska.

Both abaxial (outer) and adaxial (inner) surfaces of the sepals (Figure 5A) were glabrous and composed of regularly arranged epidermal cells with a smooth cuticle (Figure 5B). The epidermal cells towards the central part of the sepals were more elongated. Scattered stomata (guard cells ca. 25 µm in diameter, stomatal aperture ca. 10 µm in length) occurred on the adaxial surfaces (e.g., *C. rheedii* and *C. hoi*; Figure 5C). Stomata, if present, were surrounded by several shorter and somewhat concentrically arranged subsidiary cells. Most of the stomata observed produced small quantities of secretion whose residues thinly coated the guard cells (Figure 5C). The adaxial surface of the sepals possessed trichomes (Figure 5A,D). The trichomes were sunken in small depressions of the epidermis (crypts) and surrounded by several shorter and concentrically arranged cells. The cuticle of adjoining cells was not glabrous like that of other epidermal cells, but sub-parallelly wrinkled. These wrinkles or striations were more distinct and more numerous near the trichome base, where they appeared to converge. The surface of the striations was also undulate (second level of ornamentation—reported here for the first time) and coated with very small quantities of scattered secretory residues. The trichomes were small (ca. 50 µm long), erect, and bicellular: the apical cells were ovoid and relatively large, whereas the basal cells were more cylindrical and narrower (Figure 5E). On various parts of the sepal epidermis occurred narrow, sometimes branched, thread-like structures interpreted to be fungal hyphae (e.g., *C. taurinum* and *C. hoi*; Figure 5E).

#### 2.1.2. Petals

Like the sepals, both abaxial and adaxial surfaces of the petals were glabrous and composed of regularly arranged epidermal cells with a smooth cuticle. These cells were more elongated in the central part of the petal. Stomata were absent on the adaxial surfaces of the petals. Trichomes were present over the entire adaxial surfaces of the petals, or concentrated at their bases. The epidermal cells surrounding the trichomes, trichome size, cuticular ornamentation, and the type of secretions resembled those of sepals. Narrow, branched thread-like structures, presumed to be fungal hyphae, occurred on various parts of the epidermal surface.

#### 2.1.3. Labellum

**Figure 6:**
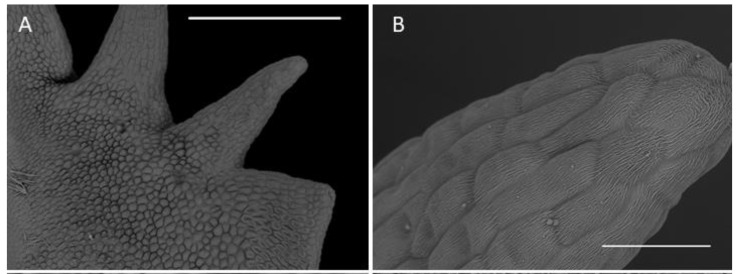

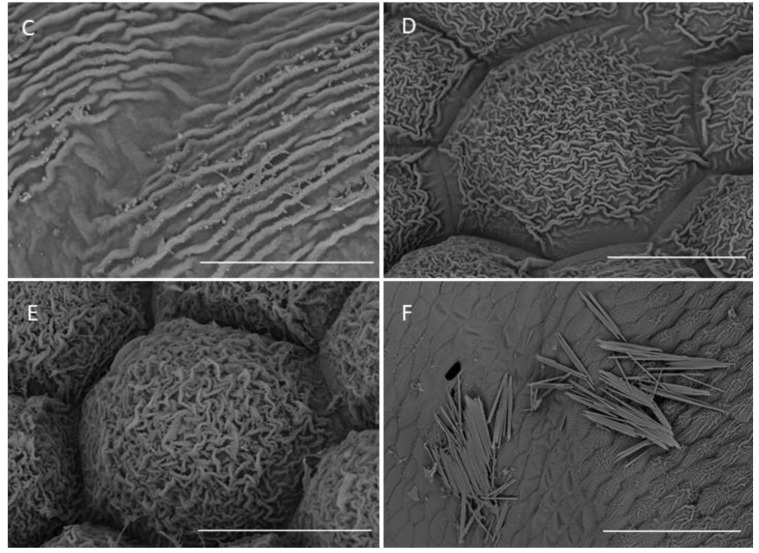
Labellar structure of *C. hoi*. (**A**)—adaxial surface of the lateral lobe (scale bar: 500 µm); (**B**)—distal part of an internal tooth of the lateral lobe showing striae (scale bar: 50 µm); (**C**)—epidermal cuticle with striae present on an internal tooth of the lateral lobe (scale bar: 10 µm); (**D**)—striate epidermal cell at base of tooth of lateral lobe (scale bar: 20 µm); (**E**)—striate epidermal cells from the upper part of lip lamina (except for auricles of lateral lobes; scale bar: 20 µm); (**F**)—epidermal cells of the labellar cavity and adjacent tissue with striate cuticle and scattered raphides (scale bar: 100 µm). Images, H. B. Margońska.

The labellum of this genus is erect (360° resupinate). The form of the labellar epidermal cells and the structure of their cuticle in the investigated *Crepidium* species were very diverse. They varied relative to their position on the adaxial surface of the labellar lamina (Figure 5F). The mid-lobe of the species studied was retuse or divided at the apex. Its distal margins and teeth consisted of elongated glabrous cells with gently curved, convex outer tangential walls. The cuticle was minutely parallelly wrinkled or striate (Figure 6B–E). The proximal part of the teeth had larger and more convex epidermal cells (Figure 6B). The epidermal cells of the proximal part of the entire mid-lobe were often much smaller and convex, and somewhat elongated. Their cuticle was finely irregularly wrinkled, especially towards the distal parts of the cells. Smaller raphide-containing cells (observed in their various stages of development) were present (Figure 6F). Small amounts of secretions were observed at the periphery of raphide-producing cells (especially during the later stages of their development).

The lateral lobes each had internal teeth (Figure 6A), except proximally, and here, the epidermal cells resembled those found on the teeth of the mid-lobe—i.e., elongated and glabrous, with gently curved, convex outer tangential walls, and finely, sub-parallel cuticular striae (Figure 6B). Furthermore, the striae were very minutely undulate (Figure 6C) and partly coated (especially on the more convex parts) with small quantities of scattered secretions. The secretions comprised minute globules (ca. 0.25 µm in diameter) and thread-like residues of viscid material. The cell walls were depressed and had a smoother cuticle, with almost no secretory residues. The external teeth of the lateral lobes resembled the teeth of the mid-lobe, except for proximally (Figure 6A), where the epidermal cells were elongate and glabrous, with gently curved, convex outer tangential walls and fine sub-parallel cuticular striae. Proximally, the teeth of the lateral lobes consisted of cells intermediate in form to those found distally and those comprising most of the labellar surface (Figure 6D). Here, the cells were polygonal, having only a slightly convex outer tangential wall with a cuticle bearing pronounced and irregularly undulate striations. Again, the distal part of the striae bore minute globules (ca. 0.25 µm in diameter) and residues of thread-like viscid material. Once more, the cell walls were depressed, and they had an almost glabrous cuticle, almost lacking any residues or secretions. Epidermal cells of a similar form occurred on most surfaces of the auricles of the lateral lobes. Only the margins of the auricles possessed epidermal cells similar in form to those found on the distal teeth of the lateral lobes (Figure 5F). Adaxially, most of the labellar lamina (except for the auricles of the lateral lobes) possessed epidermal cells of a very specific form, in that they were polygonal and apically, distinctly convex (Figure 6E). Their walls were deeply depressed. Distally, their cuticle was strongly and irregularly undulate; the striae were arranged at various heights. Once more, secretory material was very distinct, comprising both minute globules (ca. 0.25 µm in diameter) and residues of a thread-like viscid substance (Figure 6C).

#### 2.1.4. Labellar Cavity (Pseudonectary)

**Figure 7:**
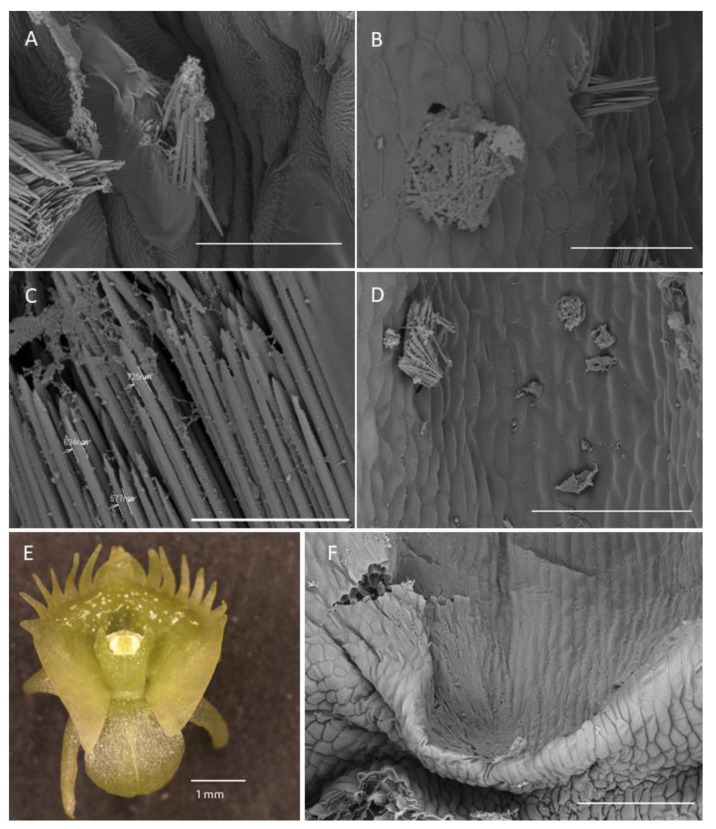
Labellar structure of *C. hoi* and *C. tixieri.* (**A**)—Raphide crystals piercing the membranes and walls of a raphide-producing cell of *C. hoi* (scale bar: 30 µm); (**B**)—release of crystals and intracellular contents (large masses of sticky material and globules) from raphide-producing cells of *C. hoi* (scale bar: 50 µm); (**C**)—raphides, sticky material and globules released from a raphide-producing cell of *C. hoi* (scale bar: 10 µm); (**D**)—cavity and adjacent tissues with raphides; sticky material and globules released from a raphide-producing cell of *C. hoi* (scale bar: 100 µm); (**E**)—raphides on the adaxial surface of a fresh flower of *C. tixieri*; (**F**)—fold covering the labellum base of *C. hoi* (scale bar: 100 µm). Images, H. B. Margońska.

**Figure 8:**
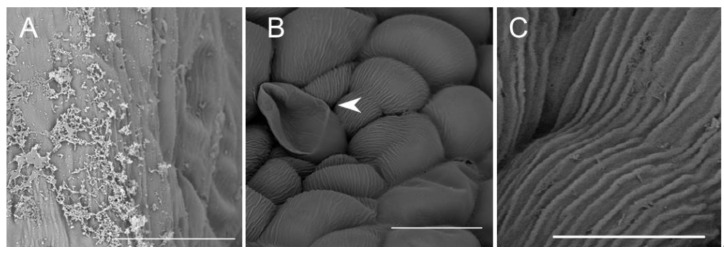
Cavity structure of *C. hoi*. (**A**)—secretory residues at bottom of labellar cavity (scale bar: 20 µm); (**B**)—bases of epidermal trichomes lining the labellar cavity with strongly undulating cuticle (scale bar: 30 µm); (**C**)—base of epidermal trichome within the labellar cavity showing an additional level of ornamentation or sculpturing of the cuticular striae (scale bar: 10 µm). Images, H. B. Margońska.

The central part of the labellar lamina has a cavity (pseudonectary) located just behind the gynostemium. Regardless of the adjacent structures, peripherally, the floor of the cavity (extending from the convex rim to the transverse semi-lunate roof at its apex), comprised epidermal cells which were more flattened, oblong, and concentrically arranged (Figure 6F). Their walls were distinctly depressed and the cuticle irregularly undulating, the sculpturing or ornamentation being more concentrated distally, where greater secretory activity was observed. Centrally, the epidermal cells lining the bottom of the cavity were somewhat irregular in shape, but usually more or less oblong, and rather flattened distally, and their walls were only gently depressed. Their striate cuticle was gently and irregularly undulating. The striae here were 2–4-fold shorter than those of the peripheral region. Secretory activity was observed to be less and more scattered (Figure 6F). Greater concentrations of raphide-containing cells were present in areas adjacent to the cavity, including its rim, roof, and floor (Figure 6F). Close examination of these cells has enabled us to trace their development, which hitherto has not been described in the literature. The sequence of development was as follows: Epidermal cells gradually began to swell and become increasingly convex. Raphides within these cells grew until they penetrated the plasmalemma and cell walls (Figure 7A,B). The crystals, which were initially arranged in bundles (Figure 7C), in time, became scattered over adjacent epidermal surfaces. The idioblastic raphide-producing cells collapsed, releasing their intracellular contents, which in scanning electron micrographs, were visible as large masses of sticky material and globules. In fresh, live flowers, they appear as shiny droplets of liquid (Figure 7D). Raphide-producing cells were also observed distally on the upper part of the gynostemium or column, especially just beneath the base of the anther and on the anther itself (Figure 7E). A small fold of tissue was present just above the base of the labellum which connected the latter to the posterior part of the gynostemium (Figure 7F). The inner surface of the fold possessed epidermal cells similar to those in other parts of the cavity. The epidermal cells present on the ridge of the fold were more or less oblong, and transversely orientated relative to the ridge. Distal cells possessed rather gently curved, convex walls—though those between adjoining cells were somewhat depressed. Sculpturing or ornamentation of the cuticle was longitudinal and somewhat more pronounced than that present on epidermal cells occurring at the bottom of the labellar cavity, but less pronounced than that on the cuticle of epidermal cells outside the cavity (Figure 7F). Further secretory residues were present at the bottom of the cavity (Figure 8A).

### 2.2. Anatomical and Histochemical Analysis

#### 2.2.1. Tepals (Petals and Sepals)

**Figure 9:**
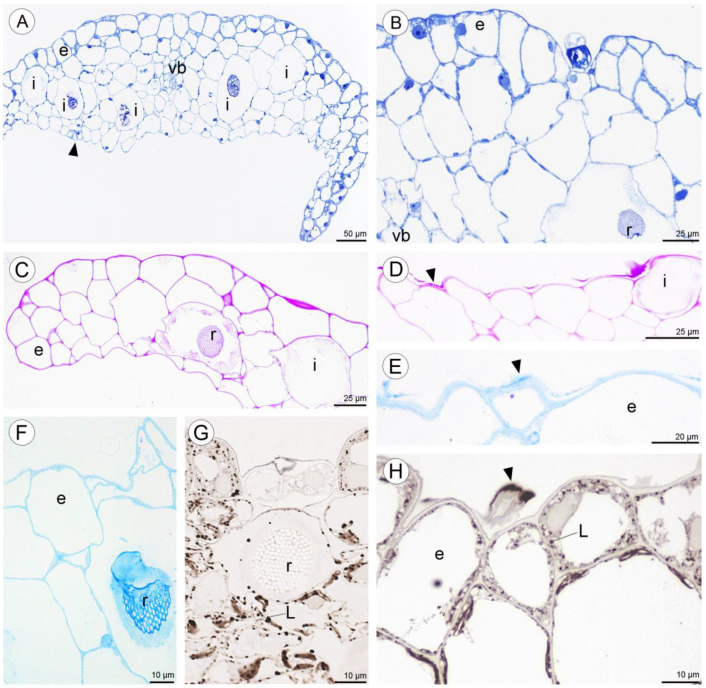

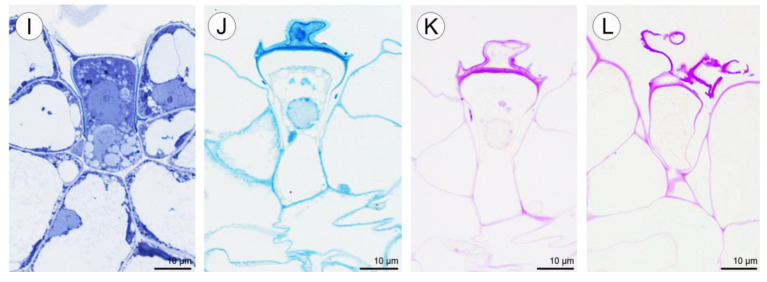
Anatomy and histochemistry of petals and sepals of *C. hoi*. (**A**)—Transverse section of petals showing a single-layered epidermis, stoma (arrowhead), vascular bundle, and idioblasts (stained with Toluidine Blue O). (**B**)—Bicellular trichome located in crypt or epidermal depression of dorsal sepal. Raphides and vascular bundle are visible (TBO); (**C**)—Transverse section through lateral sepal stained using the PAS reaction. Idioblasts with raphides occur beneath the epidermis. (**D**)—Surface material (arrowhead) stained by means of the PAS method on the surface of the lateral sepal, in close proximity to idioblasts. (**E**)—Surface protein (arrowhead) on the epidermis of lateral sepal (ABB). (**F**)—A detailed view of the epidermis, subepidermal parenchyma cells, and idioblasts with raphides of dorsal sepal stained with ABB. (**G**)—SBB stains intracellular lipid droplets in tissues of dorsal sepal. Idioblast with raphides located beneath stoma. (**H**)—Secretory material stained with SBB (arrowhead) is released onto the surface of the lateral sepal. Lipid droplets are present in epidermal cells. (**I**)—Details of the trichome of the lateral sepal (TBO). (**J**)—Trichome on the surface of the lateral sepal stained for protein (ABB). (**K**)—Trichome of the lateral sepal stained for insoluble polysaccharides (PAS). (**L**)—Section of a PAS-stained trichome. The tip of the trichome appears to be ruptured. e—epidermis, i—idioblast, L—lipid droplet, r—raphides in idioblast, vb—vascular bundle.

Anatomical results obtained during the analysis of petals and both dorsal and lateral sepals revealed the presence of stomata on the abaxial and adaxial surfaces (Figure 9A,G). In the transverse section, the ground tissue of the petals was homogeneous and contained collateral vascular bundles (Figure 9A). There were many common features visible in the transverse sections of both types of tepals, including the presence of a single-layered epidermis and large idioblasts with raphides located deeply in the parenchyma and in the subepidermal tissue (Figure 9A–C,F,G).

Positive histochemical results for proteins, lipids, and insoluble polysaccharides were obtained for both the sepals and petals. Few small individual starch grains occurred in tepal cells, but were not common (Figure 9C). However, idioblast contents consistently stained strongly for insoluble polysaccharides (Figure 9C,D). Some secretory material that stained with the PAS reaction was observed on the surface of tepals (Figure 9D), and also near stomata (not shown). Similar results were obtained using ABB, where the intensive staining of proteins was observed near bundles of idioblastic raphides and on the surface of sepals (Figure 9E,F). Lipid droplets were present in all cells of the tepals, but at relatively higher concentrations in the adaxial epidermis. Lipoidal substances were detected in small quantities on the surface of lateral sepals (Figure 9G,H). Secretions rich in protein and polysaccharides were observed close to secretory trichomes (Figure 9J–L) located in epidermal crypts (Figure 9B,I–L). Cells surrounding the base of trichomes had an undulating cuticle.

#### 2.2.2. Labellum

**Figure 10:**
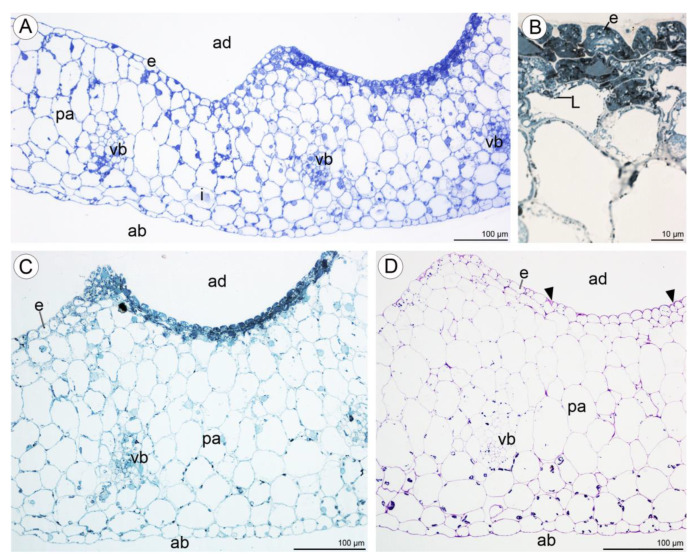
Transverse section through the lip cavity of *C. hoi*—results of histochemical tests. (**A**)—Part of the transverse section of the labellum showing the strongly stained epidermis of the labellar cavity (TBO); (**B**)—large lipid droplets accumulate in epidermal and subepidermal cells of the labellar cavity (SBB); (**C**)—epidermal and subepidermal cells of the cavity stained intensely with SBB; (**D**)—polysaccharides are present on the surface of the adaxial epidermis (arrowhead). Starch grains occurred in both epidermal and parenchyma cells (PAS). ab—abaxial surface, ad—adaxial surface, e—epidermis, i—idioblast, L—lipid droplet, pa—parenchyma, vb—vascular bundle.

Light microscopy observations of transverse, semi-thin sections (Figure 10A) revealed the presence of a single-layered epidermis covered by a cuticle and several layers of small subepidermal cells enclosing larger ground parenchyma cells. Collateral vascular bundles were embedded in the ground parenchyma. Moreover, cells of the single-layered epidermis and subepidermal tissue of the central labellum cavity stained more intensely than cells elsewhere on the lip (Figure 10A–C). The adaxial epidermal cells of the lip contained numerous lipid droplets (Figure 10B,C) and a few starch grains (Figure 10D). Some secretory material was observed on the surfaces of cells lining the cavity, and this stained for water-insoluble polysaccharides (Figure 10D). Secretory cells located in the lip cavity were visibly smaller than epidermal and parenchyma cells located elsewhere.

**Figure 11:**
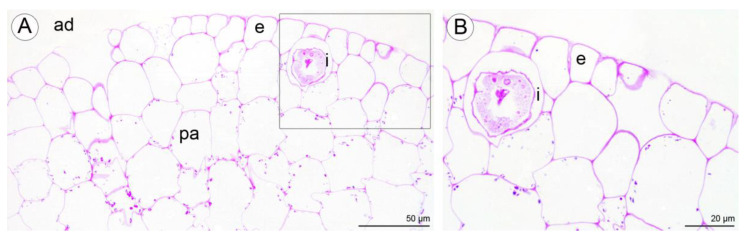
Staining of insoluble polysaccharides in labellum cells of *C. hoi*. (**A**)—Central part of transverse section of labellum showing starch grains within parenchyma cells and the presence of idioblasts in close proximity to epidermal cells (PAS). (**B**)—High magnification of the part of image **A** showing an idioblast (PAS). ad—adaxial surface, e—epidermis, i—idioblast, pa—parenchyma.

**Figure 12:**
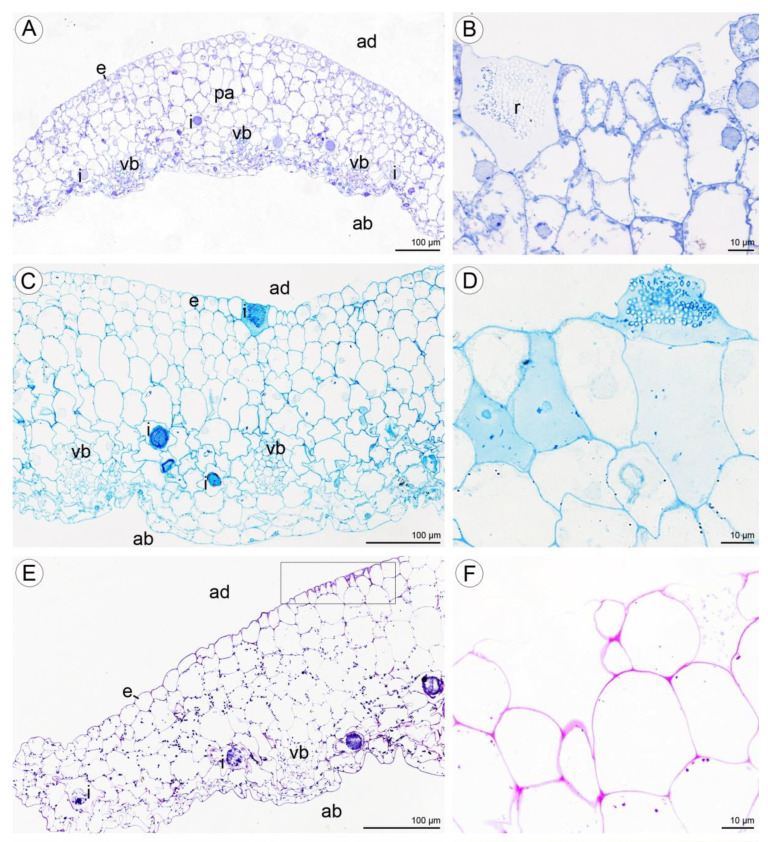

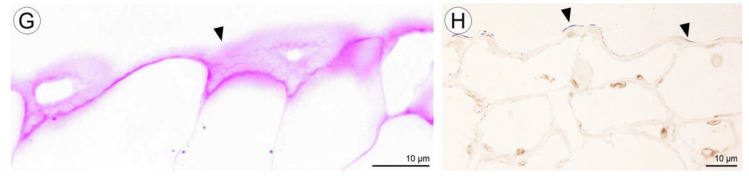
Histochemical features of lip of *C. hoi*. (**A**)—Transverse section of the lip showing epidermis, parenchyma cells, vascular bundles, and idioblasts (TBO); (**B**)—idioblasts with raphides located between epidermal cells of adaxial lip surface (TBO); (**C**)—transverse section through the lip with strongly stained idioblasts (ABB); (**D**)—a detailed view of the adaxial surface of the lip with idioblasts stained strongly for proteins (ABB); (**E**)—a transverse section through the lip stained for insoluble polysaccharides (PAS); (**F**)—idioblasts located between the epidermal cells of the adaxial surface of the lip following release of raphides (PAS); (**G**)—a high power view of the area outlined in (**E**) showing the outer tangential walls of the adaxial epidermis of the lip. Polysaccharide secretion appears to be associated with the presence of micro-channels in the cuticle (arrowhead, PAS); (**H**)—lipid material on adaxial surface of the lip (arrowheads, SBB). ab—abaxial surface, ad—adaxial surface, e—epidermis, i—idioblast, pa—parenchyma, r—raphides in idioblast, vb—vascular bundle.

Numerous large raphide-containing idioblastic cells were distributed throughout the tissues of the labellum, including the subepidermal and ground parenchyma (Figure 11A,B and Figure 12A,C,E). The idioblasts near the abaxial surface were always hypodermally located. However, adaxially, idioblasts also occurred between epidermal cells (Figure 11A,B and Figure 12B–D,F), and raphides could sometimes be seen between the outer tangential wall of epidermal idioblasts and the cuticle (Figure 12D). Some adaxial epidermal cells both within and outside the labellar cavity seemed to have discharged their contents, which stained both for protein (Figure 12C,D) and insoluble polysaccharides (Figure 12E,F), together with raphides, onto the surface of the labellum. The chemical content of idioblasts was identical, regardless of location.

**Figure 13:**
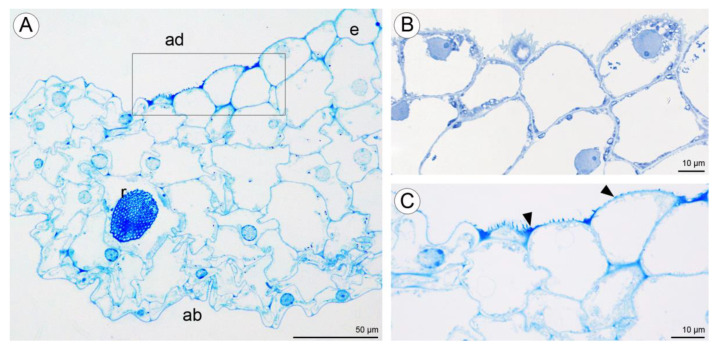
Histochemical features of lip of *C. hoi*. (**A**)—Transverse section of the lip (ABB); (**B**)—detail of adaxial epidermal cells with undulated cuticle (TBO); (**C**)—a high power view of area of the adaxial epidermis outlined in **A** that was strongly stained for proteins (arrowheads, ABB). ab—abaxial surface, ad—adaxial surface, e—epidermis, r—raphides in idioblast.

Histochemical tests revealed the presence of unevenly distributed starch grains in all cells of the lip (Figure 12E,F). Polysaccharide secretion appeared to be associated with the presence of micro-channels in the adaxial cuticle, where insoluble polysaccharides were detected (Figure 12G). Some secreted lipoidal material was also observed on the lip’s surface, where it formed a thin layer (Figure 12H). Proteins were present adaxially near the margins of the lip, where the cuticle was more expanded (Figure 13A–C). However, the individual parts of the three-lobed labellum showed significant differences at the histochemical level.

### 2.3. Ultrastructural Analysis (TEM)

#### 2.3.1. Tepals (Petals and Sepals)

**Figure 14:**
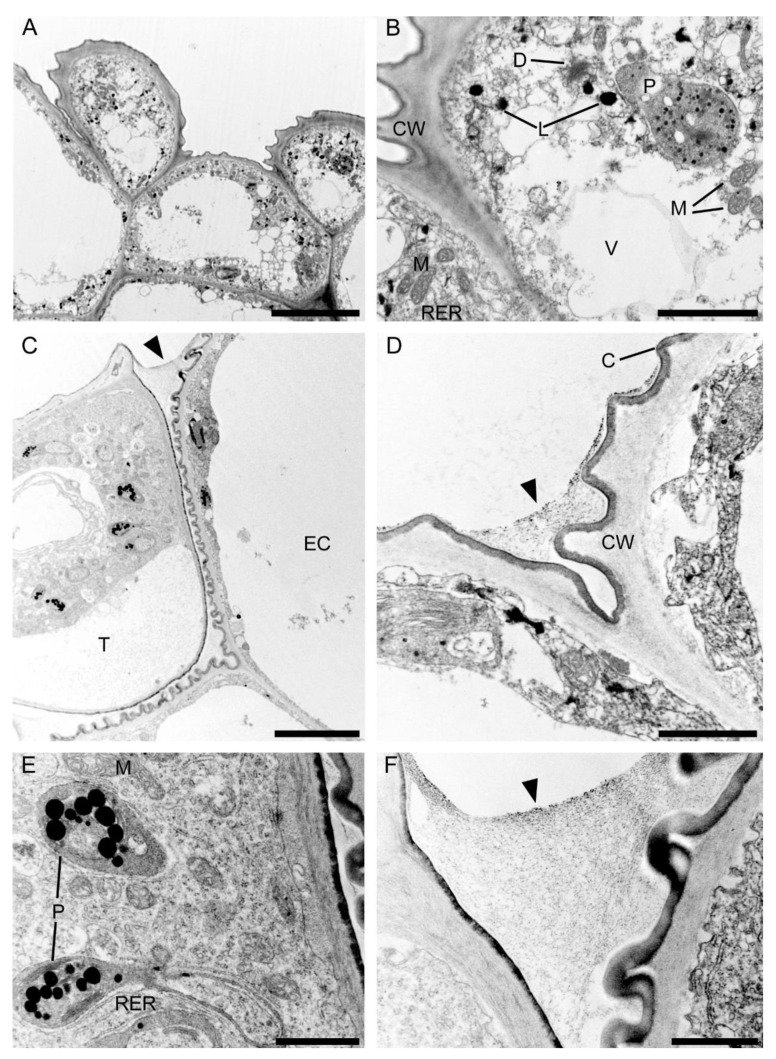
Ultrastructure of the tepals of *C. hoi*. (**A**)—Highly vacuolate cells of single-layered adaxial epidermis (scale bar: 8 µm). (**B**)— A detailed view of **A**, showing the cytoplasm with mitochondria, plastids containing a few starch grains, and numerous plastoglobuli; profiles of rough endoplasmic reticulum, vacuoles, lipid droplets, and dictyosomes (scale bar: 2 µm). (**C**)—Secretory trichome with dense cytoplasm, numerous organelles and associated surface material (arrowhead) (scale bar: 6 µm). (**D**)—Base of the trichome, the cell wall possessing an undulate cuticle with secreted surface material (arrowhead) (scale bar: 1 µm). (**E**)— A detailed view of **C**, showing dense cytoplasm containing plastids with a few plastoglobuli, and mitochondria and profiles of rough endoplasmic reticulum (scale bar: 1 µm). (**F**)—Interface of trichome and epidermal cell, the cavity between them containing copious secreted material (arrowhead) (scale bar: 0.8 µm). C—cuticle, CW—cell wall, D—dictyosome, EC—epidermal cell, L—lipid droplet, M—mitochondrion, P—plastid, RER—rough endoplasmic reticulum, T—trichome, V—vacuole.

Observations by means of transmission electron microscopy revealed that the single-layered adaxial epidermis of the sepals consisted of highly vacuolated cells (Figure 14A). The cytoplasm of these cells contained mitochondria, plastids with few starch grains and large numbers of plastoglobuli, profiles of rough endoplasmic reticulum, vacuoles, and lipid droplets. Dictyosomes were observed sporadically (Figure 14B). The cell wall possessed an undulate cuticle upon which small quantities of secretion were present (Figure 14C). Secretory trichomes on both the adaxial and abaxial surface of the sepals were located in localized epidermal crypts. Such trichomes contained dense cytoplasm with cup-shaped plastids containing relatively large electron-dense plastoglobuli. Mitochondria, profiles of rough endoplasmic reticulum, and large and small vacuoles containing fibrillar material were also present (Figure 14D,E). At the base of each trichome, within the epidermal crypt, a strongly undulate cuticle was present (Figure 14D), and this supports SEM observations (Figure 8B). Ultrastructurally, at the interface between trichome and epidermal cells, copious intercellular secreted material was present. This secretion was observed throughout the entire epidermal crypt (Figure 14D,F).

#### 2.3.2. Labellum

**Figure 15:**
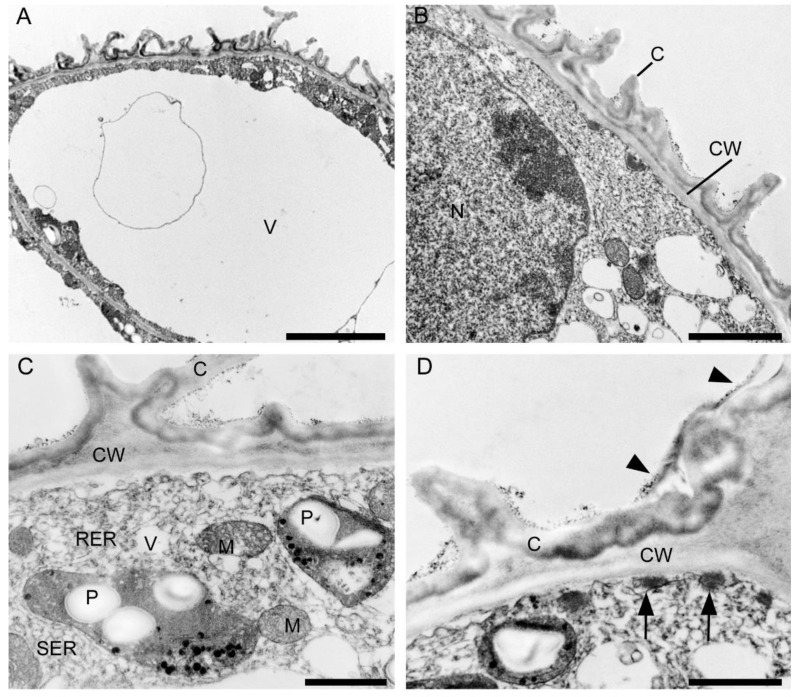

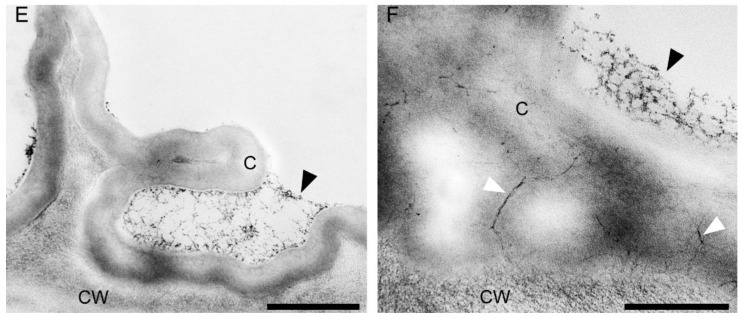
Ultrastructural analysis of the adaxial surface of the lip of *C. hoi.* (**A**)—An epidermal cell containing a single vacuole (scale bar: 6 µm). (**B**)—A detailed view of an epidermal cell with a large nucleus and thick cell wall with an undulating cuticle (scale bar: 2 µm). (**C**,**D**)—An epidermal cell at higher magnification. (**C**)—Dense cytoplasm containing plastids with starch grains and plastoglobuli, together with mitochondria, smooth and rough endoplasmic reticulum, and vacuoles. The cell wall here is lamellate and has an undulate cuticle (scale bar: 1 µm). **D**—The striate cell wall bears small amounts of secreted material (arrowheads) and electron-dense bodies are present close to the irregular plasmalemma (arrows) (scale bar: 1 µm). (**E**)—The thick, striate cell walls possess a cuticle bearing small amounts of secreted material (arrowhead) (scale bar: 0.6 µm). (**F**)—A detailed view of E showing micro-channels in the cuticle (white arrowheads), and a little secreted material (black arrowhead) (scale bar: 0.4 µm). C—cuticle, CW—cell wall, M—mitochondrion, P—plastid, RER—rough endoplasmic reticulum, SER—smooth endoplasmic reticulum, V—vacuole.

Ultrastructural analysis of the adaxial epidermis of the labellum revealed that its cells contained a large central vacuole, occasionally with intravacuolar flocculent material (Figure 15A). The parietal cytoplasm contained a large nucleus, mitochondria, smooth and rough endoplasmic reticulum, small vacuoles, and plastids with starch grains and plastoglobuli (Figure 15B,C). Dictyosomes and lipid droplets, however, were very seldom seen. The plasmalemma was irregularly folded and had small invaginations (Figure 15C,D). Moreover, electron-dense, osmiophilic bodies located close to the irregular plasmalemma structurally resembled tannin-like material (Figure 15D). The thick outer tangential wall of adaxial epidermal cells of the labellum occasionally appeared lamellate (Figure 15C), though remarkably, the cell walls of both the labellum and tepals possessed hardly any primary pit-fields or plasmodesmata (Figure 14 and Figure 15). A heterogeneous cuticle (Figure 15B–D) with small amounts of secreted material was present adaxially on the thick outer tangential cell wall of the labellum (Figure 15D,E) and it contained micro-channels (Figure 15F).

### 2.4. Chemical Analysis

#### The Chemical Composition of Sugar Fraction and Surface Lipids

An overview of the results for sugar analysis is given in Table 1. Agueous extracts of the flowers of *C. hoi* contained a sugar mixture composed of fructose and glucose, suggesting the presence of hexose-dominant nectar directly available to pollinators. All the other species produced sucrose-rich or sucrose-dominant nectar. The chemical composition of methanolic extracts obtained from the same samples was usually more balanced in terms of sucrose/hexose ratio, which clearly shows differences in the chemical composition of the nectar secreted. Fewer sugars can be extracted from the plant tissues. The only exception was *C. luniferum*, where nectar was characterized by a slight dominance of hexoses, whereas the methanolic extract was composed of sucrose only.

**Figure 16:**
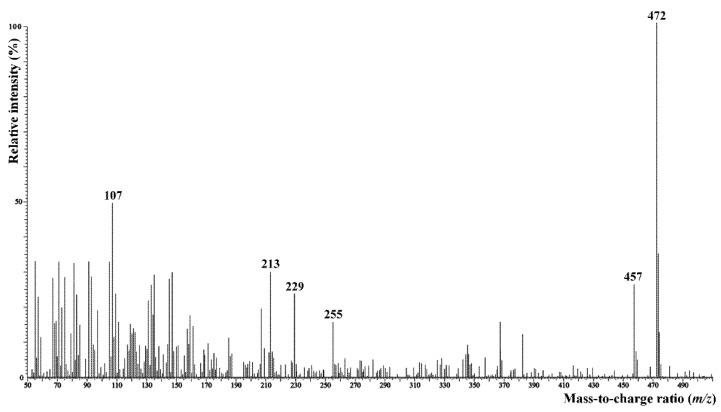
Mass spectrum (EI, 70 eV) of 5α-ergost-8(14)-en-3β-ol isolated from *C. hoi* flower surface lipids. Signal intensity in relation to the base peak (*m*/*z* 472) intensity.

Surface lipids of *C. hoi* were composed mostly of saturated and unsaturated hydrocarbons C16–C31 (46%), followed by significant amounts of free glycerol and aliphatic alcohols (33%), sterols (13%), and fatty acids as a minor fraction (3%). The sterol fraction contained not only standard phytosterols, but also small amounts of the much less common 5α-ergost-8(14)-en-3β-ol, which was identified based on its mass spectrum (Figure 16) obtained from GC–MS analysis [8]. A similar lipid composition, but with a significantly higher proportion of fatty acids, was also observed in *C. acutangulum*. The amounts of some fatty acids, however, may possibly have been overestimated in samples or when extracted from tissues. Hydrocarbons were much less abundant in lipids of *C. luniferum*. Atypical hydrocarbons of medium chain length (C14–C18) and an even number of carbon atoms was detected in all plants. Very long-chain fatty acids (C30–C32) were rarely detected as minor components in the lipids of *C. luniferum*. An overview of the results is given in Table 2.

## 3. Discussion and Conclusions

Traditionally, representatives of the genus *Crepidium* were considered not to offer any floral food reward to their pollinators, but instead were thought to attract them solely by means of visual stimuli (mainly color) ([1] and references therein). However, as our results demonstrate, these assumptions were wrong.

Stomata occur on the surfaces of the sepals irrespective of the size of the flowers and their characteristics. *Crepidium hoi, C. luniferum, C. tixierii*, and *C. taurinum,* with rather small flowers for the genus (ca. 0.42–0.95 cm, see Table 3), have numerous stomata on their sepals, whereas *C. acutangulum* and *C. resupinatum* have significantly larger flowers (ca. 0.80–1.20 cm), but much fewer stomata. Stomata on petals are much rarer. Here, there appears to be a correlation between the presence of stomata and the shape of the petals, regardless of petal size in general, with stomata occurring more frequently on wide petals (obovate, ovate, or oblanceolate, but not on lanceolate or linear petals). The stomata were associated with several shorter and somewhat concentrically arranged subsidiary cells. For some species, such as *C. hoi*, *C. rheedii*, and *C. taurinum*, we were able to confirm that stomata produced small amounts of secretion. TEM studies and staining with PAS confirmed the presence of this secretion in close proximity to stomata on the adaxial epidermal surfaces of the dorsal sepals of all investigated species.

Trichomes commonly occur on the sepals of Malaxidinae and Liparidinea [1]. They are located in small depressions of the epidermis known as crypts and surrounded by several shorter and somewhat concentrically arranged epidermal cells. These cells have a much-sculptured cuticle, which is concentrically folded relative to stomata. Our SEM results demonstrated for the first time the presence of an additional level of ornamentation on the striae, resulting in their undulate appearance (e.g., *C. hoi*, Figure 8C). The surfaces of these striae were coated with very small and scattered residues of secretion. Trichomes were rare on the adaxial surfaces of petals. We observed, however, that they are dispersed over the entire surface of wide petals (e.g., *C. hoi*) or towards the base of narrow or linear petals (e.g., *C. rheedii*, *C. resupinatum*, *C. tixieri*, and *C. taurinum*). TEM investigation indicated that the dense cytoplasm of the trichomes of all *Crepidium* species investigated contained abundant organelles indicative of high metabolic activity. These included numerous mitochondria, and plastids containing large numbers of plastoglobuli, but starch grains were absent. Profiles of rough endoplasmic reticulum, and large and small vacuoles, were also present. The roles of these trichomes are not completely clear, but they may perhaps serve as a means of distributing secretions.

Results obtained for histochemical, ultrastructural, and chemical analyses are compatible and demonstrate the presence of secreted substances on the surface of *Crepidium* flowers. Polysaccharides were observed on the surface of the labellar cuticle, and on other tepals, but in very small quantities. Secretory activity appears to be relatively greater on the lip than in sepals and petals of *Crepidium*. This statement is further supported by stereoscopic microscopy, which revealed the presence of discernible, small, translucent droplets on the adaxial lip surface. These droplets were present in all species investigated, but in small numbers, which is consistent with observations made on representatives of tribe Malaxideae [9].

TEM studies of the lip cavity of *C. hoi* confirmed that epidermal cells are involved in secretion. These have striated cell walls whose cuticle contains micro-channels and is coated with small quantities of secreted material. The cytoplasm again contains abundant organelles indicative of high metabolic activity. Micro-channels present in the cuticle overlying the labellum (e.g., *C. hoi*) may facilitate secretion and the resorption of secreted material. The dense cytoplasm contains both smooth and rough endoplasmic reticulum, and mitochondria, whereas the plastids contain starch grains and plastoglobuli. Similar organelles have been observed in the nectary spur of *Anacamptis pyramidalis* f. *fumeauxiana* Marg. & Kowalk. ([10] and references therein), and were confirmed to play a role in the synthesis of volatile substances. The hydrolysis of starch results in a source of energy for fragrance and nectar production [11,12]. The volatile components of fragrances are synthesized in plastoglobuli and are probably transferred from them to the intraplastidal membranes, before traversing the plastid envelope, passing to the endoplasmic reticulum, and finally traversing the plasmalemma [13,14]. In the labellum, electron-dense material was observed to accumulate in close proximity to the irregular plasmalemma and structurally resembled that of tannin-like material. A similar substance has been described for *Epipogium aphyllum* Sw. [15]. Such polyphenolic biomolecules are known to function as a protective barrier against pathogens, herbivores, and ultraviolet radiation [16].

Histochemical tests also confirmed elevated levels of insoluble polysaccharides and proteins within idioblasts, as compared with other epidermal and parenchyma cells. Idioblasts with raphides of calcium oxalate occur in all tepals, mainly in the subepidermal and ground parenchyma, but adaxially, they may also occur within the epidermis. Enlargement and rupture of epidermal idioblasts, owing to the growth of raphides, as has been previously described for other representatives of Orchidaceae [17,18], releases the latter onto the lip surface. Raphides may also play a role in attracting pollinators by gathering and reflecting light ([19] and references therein). This may be further enhanced in that epidermal idioblasts and released bundles of raphides may stand proud of the otherwise relatively smooth lip surface and are thus more prominent, and also because of the reflective qualities of the labellar secretion, both of which may contribute to increased reproductive success by compensating for the size of small flowers and increasing their visibility to pollinating insects [18,20,21].

Raphide-producing cells were more concentrated in areas adjacent to the labellar cavity—in particular, its rim, roof, and floor. It is well known that such cells frequently occur in the epidermis of representatives of the genus *Crepidium* [1]. That raphides consist of calcium oxalate was confirmed by means of chemical tests. The presence of raphide-producing cells both within the epidermis and deeper in the subepidermal tissues was reported here for the first time and has been confirmed by TEM. Such cells are easy to distinguish from those of other tissues, regardless of size or stage of development, owing to the different nature of their cytoplasm and their crystalline content. Observations of these cells have allowed us to trace their development for the first time. The first stages (initial stages of crystal cluster formation) were most frequently observed in subepidermal tissues. Gradually, as the cells expand and undergo changes to the composition of their cytoplasm, together with concomitant development of crystals, the idioblasts are displaced closer to the epidermis, whereupon the outer tangential walls become increasingly convex (Figure 3B and Figure 12D), and at this stage they appear as shiny areas on the surface of living tepals (Figure 3A). As the raphides grow, they pierce the plasmalemma and cell wall. Eventually, the cuticle is ruptured too, and the crystals, which are initially grouped in bundles, with time, become scattered over the tepal surface, whereupon, in live tepals, they reflect light and glow intensely (Figure 6E). Similarly, as the idioblasts collapse, the cell contents are discharged onto the tepal forming minute, shiny droplets of liquid that resemble nectar. Furthermore, penetration of the cell wall by raphides may also release volatiles, which may provide an olfactory cue, and together with the nectar-like droplets, attract potential insect pollinators. Individual raphides released from ruptured raphide-producing cells were particularly strongly fluorescent under UV light, and since many insects are able to see at this wavelength, they may provide a visual cue for pollinators. This is further enhanced by their concentration in structures involved in the pollination process, such as the anther, the central part of the labellum, especially near the lip cavity, and localized behind the gynostemium, where by reflecting light, they may play an important role in guiding insects into the flower.

The results of chemical analyses are summarized in Table 2. The possible biological role of floral surface lipids may include protection from both abiotic and biotic factors. Saturated hydrocarbons are considered to be the most effective barrier against non-stomatal water loss (cuticular transpiration) [22], whereas some unsaturated hydrocarbons, including nonacosene, which was detected in most of the samples, have already been identified as contact sex pheromones in some insects [23]. The presence of small amounts of medium-chain C9–C12 saturated fatty acids perhaps indicates the importance of surface lipids in preventing fungal infections, since these compounds have previously been described as effective inhibitors of phytopathogenic fungal growth [24].

Of the several dozens of *Crepidium* species cultivated and investigated, hitherto, there have been no reports of floral fragrances perceptible to humans. We have, however, now detected them in certain species (e.g., *C. luniferum* sect. *Crepidium* which produces a honey-like scent), both in situ and ex situ. Compared with other species that have been analyzed, this particular taxon displayed the highest proportions of other compounds such as sucrose in methanolic sugar extracts and many fatty acids in floral surface lipids.

It is difficult to state exactly what the effective olfactory compounds are based solely on chemical analyses. What is certain is that volatile compounds are present. Micromorphological observations confirm that nectar *sensu stricto* is absent in *C. luniferum*; however, copious surface secretions are produced by the floral epidermis and possibly by the floral trichomes. In the absence of true nectar, the honey-like scent may yet be another case of mimicry, although the potential pollinators may perhaps still be able to use the surface secretions of sepals as a rich source of polysaccharides and protein. Whether this is actually the case requires further investigation in the field.

## 4. Materials and Methods

The present research aimed to investigate the floral morphology and micromorphology of representatives of the genus *Crepidium* (Malaxidinae). Prior to selecting species suitable for this study, dozens of taxa were examined. The main criterion for choosing the material investigated in this paper was maximum floral diversity relative to the range of pollination processes in the selected section (i.e., diameter, color, color changes, presence or absence of fragrance, etc., in flowers compared with perception, size, and habits of pollinators). Thus, the following eight species were chosen: *C. acutangulum*, *C. hoi*, *C. luniferum*, *C. resupinatum*, *C. rheedii*, *C. metallicum*, *C. taurinum*, and *C. tixierii* (Table 3).

Observations began in 2001, both in situ (e.g., during the first and corresponding author’s expeditions to Indonesia and Oceania—*C. acutangulum*, *C. luniferum*, *C. resupinatum*, and *C. rheedii*) and/or ex situ (by the first author—e.g., *C. acutangulum*, *C. hoi*, *C. luniferum*, *C. metallicum*, *C. taurinum*, and *C. tixierii*).

The plants were cultivated by the first author in greenhouses at the University of Gdańsk (Poland), where they flowered regularly each season. The flowering period usually lasted about 3 to 5 weeks. Individual flowers lasted well on their inflorescences, each of the latter bearing (10)20–80 flowers, depending on species, for an average of 2–5 days. Specimens of the cultivated species were regularly photographed and collected by the first author at each stage of anthesis, and pressed and dried for herbarium sheets or preserved in Copenhagen mixture (collection: UGDA-HBM).

Observations of gross floral morphology were carried out using a stereoscopic microscope. Chemical, SEM, TEM, and histochemical analyses were performed on young, but fully developed flowers collected prior to early morning water sprinkling.

The identification of plant material was confirmed by the first author based on classical methods of taxonomy, and by referring to the original material, such as type-specimens and protologues.

### 4.1. Light Microscopy and Histochemical Analysis

For light microscopy observations, flowers were fixed for 4 h at room temperature in a mixture of 2.5% (*v*/*v*) glutaraldehyde and 2.5% (*v*/*v*) formaldehyde (obtained from paraformaldehyde) in 0.05 M cacodylate buffer pH 7.0. After fixation, the plant material was rinsed with cacodylate buffer, then dehydrated in a graded acetone series, and finally embedded in Spurr’s resin. Semi-thin (0.5–1.5 μm) transverse sections were prepared using a Sorvall MT 2B ultramicrotome and glass knives, and mounted on glass microscope slides.

For histochemical analyses, semi-thin control sections for light microscopy were stained with 0.05% (*w*/*v*) alcoholic Toluidine Blue O (TBO). Detection of protein and water-insoluble polysaccharides was undertaken using Aniline Blue Black (ABB) and the periodic acid-Schiff reaction (PAS), respectively [25]. Moreover, sections of plant material prepared for TEM were treated with Sudan Black B (SBB) to detect the presence of lipids [26].

Observations and photographic documentation were undertaken using a Nikon Eclipse E 800 light microscope equipped with a Nikon DS-5Mc camera with Lucia Image software (University of Gdańsk, Gdańsk, Poland).

### 4.2. Scanning Electron Microscopy (SEM)

Samples for SEM were fixed at room temperature in 2.5% (*v*/*v*) glutaraldehyde and 2.5% (*v*/*v*) formaldehyde in 0.05M cacodylate buffer pH 7.0, dehydrated using an ethanol series, subjected to critical point drying using liquid CO_2_, coated with gold, and examined using a Hitachi Tabletop Microscope TM-1000 at an accelerating voltage of 15 kV.

### 4.3. Transmission Electron Microscopy (TEM)

For TEM studies, floral material was fixed in a mixture of 2.5% (*v*/*v*) glutaraldehyde and 2.5% (*v*/*v*) formaldehyde in 0.05 M cacodylate buffer pH 7.0, at room temperature, for 4 h. It was then rinsed in the same buffer and post-fixed in 1% (*v*/*v*) osmium tetroxide in cacodylate buffer overnight at 4 °C. The samples were treated with 1% (*w*/*v*) uranyl acetate for 1 h, dehydrated in an acetone series, and embedded in Spurr’s resin. Ultra-thin sections (70–90 nm) were cut with a diamond knife using a Leica EM UC7 ultramicrotome and then post-stained with a saturated solution of uranyl acetate in 50% ethanol and 0.04% lead citrate [27]. The sections were examined using an FEI Tecnai G2 Spirit TWIN/BioTWIN transmission electron microscope at an accelerating voltage of 120 kV at the Laboratory of Electron Microscopy/Faculty of Biology/University of Gdańsk (Poland).

### 4.4. Epifluorescence Analysis

Autofluorescence of flowers (using UV-2A, B-2A and G-2A filters) was observed by means of an upright Nikon ECLIPSE E 800 fluorescence microscope equipped with a DS-5mc digital color microscope camera and Nikon NIS-Elements software suite.

### 4.5. Chemical Analysis

Pyridine, *N,O*-bis(trimethylsilyl)trifluoroacetamide (BSTFA) + 1% trimethylchlorosilane (TMCS) and D-(+)-glucose were purchased from Sigma-Aldrich Poland (Poznań, Poland); D-(−)-fructose was obtained from Merck KGaA (Darmstadt, Germany). Hydroxylamine hydrochloride and dichloromethane were purchased from POCH S.A. (Gliwice, Poland), sucrose from Stanlab (Lublin, Poland), and methanol from Chempur (Piekary Śląskie, Poland).

Flowers were subjected to sequential solvent extraction. Firstly, water-soluble sugars were isolated by dipping flowers in 10 mL distilled water for 30 s. Excess water was gently removed using filter paper, and volatiles and lipids subsequently extracted in 10 mL dichloromethane for 30 s. Finally, the remaining sugars (i.e., those not present directly on a flower’s surface) were extracted by dipping flowers for 15 s in 10 mL methanol. Aqueous extracts were diluted with ca. 20 mL methanol and the solvents evaporated under reduced pressure. The procedure was repeated until no water remained in the extract. Methanolic sugar extracts and dichloromethane extracts were concentrated under reduced pressure to ca. 1 mL. Extracts were then kept at 4 °C until they were analyzed.

Sugars extracted using water and methanol were subjected to a two-step derivatization procedure according to the slightly modified method described by Ruiz-Matute et al. [28]. Firstly, oximes were synthesized by adding 0.1 mL of a 2.5% hydroxylamine hydrochloride solution in pyridine. Oximes obtained in this way were transformed to respective trimethylsilyl (TMSi) derivatives by adding 0.1 mL BSTFA + TMCS (99:1). Each reaction was performed at 70 °C for 30 min. Derivatives were then analyzed using gas chromatography with a flame ionization detector (GC-FID). The analysis was performed using a Clarus 500 gas chromatograph (Perkin-Elmer Instruments, Waltham, MA, USA), equipped with a 30 × 0.25 mm i.d., film thickness 0.25 μm, Zebron ZB-5 capillary column (Phenomenex, Torrance, CA, USA). The column temperature was programmed to run from 80 °C to 300 °C at a rate of 4 °C min^−1^. Injector and detector temperatures were both set to 320 °C. Argon was used as the carrier gas at a flow rate of 1 mL min^−1^. The split ratio was 1:20, and the injection volume was 1 μL. Identification of compounds was based on retention times, which were compared with those of the analytical standards for glucose, fructose, and sucrose analyzed under the same conditions. The relative composition of the fraction was calculated based on the peak area of each compound.

Surface lipids were analyzed using gas chromatography–mass spectrometry (GC–MS). All GC–MS analyses were performed using a Shimadzu QP-2010SE system (Shimadzu, Kyoto, Japan), equipped with a 30 × 0.25 mm i.d., film thickness 0.25 μm, Zebron ZB-5MS capillary column (Phenomenex). Helium was used as the carrier gas at a flow rate of 1 mL min^−1^. Electron ionization (electron energy 70 eV, ion source temperature 200 °C) was used. Surface lipids, except for hydrocarbons, were transformed to the corresponding TMSi derivatives, which were synthesized following the removal of dichloromethane under a gentle stream of nitrogen by adding 0.1 mL BSTFA + TMCS (99:1). The reaction was performed at 90 °C for 30 min. Derivatives were then analyzed using GC–MS under the following conditions: the split ratio was 1:20, and the injection volume was 1 μL; the injector and GC–MS interface temperatures were held at 310 °C. The column temperature was programmed to run from 80 °C to 310 °C at a rate of 4 °C min^−1^ and held at 310 °C for 5 min. Compounds were identified based on their mass spectra, and the relative composition of each fraction was calculated based on the peak areas of the compounds therein.

## Figures and Tables

**Table 1:** The relative composition (%) of the sugar fraction obtained by extraction using water and methanol; nd—not detected.

Compound	*C. hoi*		*C. acutangulum*		*C. luniferum*	
	Water	Methanol	Water	Methanol	Water	Methanol
Fructose	53.82	34.76	27.27	39.07	29.22	Nd
Glucose	46.18	25.49	3.97	15.05	29.78	Nd
Sucrose	Nd	39.75	68.75	45.89	41.00	100.00

**Table 2:** The relative composition (%) of floral surface lipids in the plant species studied; (*) possibly overestimated.

Compound	*C. hoi*	*C. acutangulum*	*C. luniferum*
Glycerol	11.40	13.44	11.45
Octadecanol	0.12		
Tetracosanol	3.08		0.75
Pentacosanol	0.40		
Hexacosanol	5.12		
Heptacosanol	0.75		
Octacosanol	7.24		0.68
Nonacosanol	0.68		
Triacontanol	4.37		1.64
**Total alcohols**	**33.16**	**13.44**	**14.52**
Nonanoic acid	0.38	1.92	1.47
Decanoic acid	0.11	0.53	
Dodecanoic acid	0.15		
Tetradecanoic acid	0.37	0.29	0.37
Pentadecanoic acid	0.18		0.47
Hexadecanoic acid*	1.16	15.62	22.06
Octadecadienoic acid	0.25	0.77	2.28
Octadecaenoic acid		0.74	2.82
Octadecanoic acid*	0.35	26.73	20.04
Tetracosanoic acid			0.60
Octacosanoic acid			1.13
Triacontanoic acid			2.84
Dotriacontanoic acid			1.26
**Total fatty acids**	**2.93**	**46.61**	**55.35**
Tetradecane		1.48	1.47
Pentadecane			
Hexadecane	0.71	2.03	1.49
Octadecane	0.65	1.50	1.08
Eicosane	0.31		
Docosane	0.41		
Tricosene	1.17		0.90
Tricosane	0.93	1.72	0.69
Tetracosane		1.03	
Pentacosene	0.69	0.79	0.32
Pentacosane	4.29	3.38	1.01
Hexacosane	1.37	1.78	0.67
Heptacosene	1.60	0.47	0.58
Heptacosane	14.56	10.75	2.77
Octacosane	2.61	2.83	1.76
Nonacosene	1.70	1.25	1.43
Nonacosane	13.58	5.40	6.00
Triacontane	0.63		
Hentriacontane	0.72		
**Total hydrocarbons** ***(saturated*** *)* ***(unsaturated)***	**45.93** ***40.77*** ***5.16***	**34.41** ***31.89*** ***2.51***	**20.18** ***16.95*** ***3.22***
Campesterol	3.62		1.12
5α-ergost-8(14)-en-3β-ol	0.86		
Stigmasterol	1.03	1.62	4.66
Sitosterol	2.97	3.93	2.50
Sitostanol	4.37		
24-Methylenecycloartanol			1.68
**Total sterols**	**12.85**	**5.55**	**9.96**
Unidentified	5.12		

**Table 3:** Summary of the characteristics of the research material.

	*Species of Crepidium*							
Character	*C. acutangulum*	*C. hoi*	*C. luniferum*	*C. resupinatum*	*C. rheedii*	*C. metallicum*	*C. taurinum*	*C. tixierii*
Where observed	in situ ex situ	in situ	in situ ex situ	in situ ex situ	in situ ex situ	in situ ex situ	in situ	in situ
Diameter of flowers (cm)	0.80–1.20	ca. 0.60	0.47–0.56	0.80–1.20	0.50–0.70	0.90–1.40	0.55–0.95	0.42–0.55
Relative size of flowers for genus	large	Small	small	large	small	large	medium	small
Main colour of mature, adult flower	white	yellow, pink-purple centrally	white	dark red to maroon, violet	purple to purple-red	cream-yellow to purple	yellow, ochre, reddish to purplish	green
Scent perceptible to humans	−	−	+	−	−	−	−	−
Labellar sectretion	+	+	+	+	+	+	+	+

(+) present/detected, (−) not present/not detected.

## Data Availability

Voucher samples are deposited in the UGDA-HBM collection and are available upon request.
